# Occupational therapy students’ and educators’ perspectives and understanding of the role of occupational therapy within social prescribing: A qualitative interview study

**DOI:** 10.1177/03080226241270442

**Published:** 2024-08-13

**Authors:** Siobhan Elliott, Catherine Haighton

**Affiliations:** 1 Department of Social Work, Education and Community Wellbeing, Faculty of Health and Life Sciences, Northumbria University, Newcastle upon Tyne, UK; 2 Bradford District Care NHS Foundation Trust, New Mill, Saltaire, Shipley, UK

**Keywords:** Occupational therapy students, occupational therapy educators, social prescribing, qualitative

## Abstract

**Introduction:**

Social prescribing is building traction internationally and within the United Kingdom healthcare system, highlighted as the biggest investment in the National Health Service. The Royal College of Occupational Therapists has highlighted the contribution that occupational therapy can make to social prescribing. Therefore, this study aimed to investigate Bachelor of Science, Master of Science and Degree Apprenticeship Occupational Therapy students’ and educators’ perspectives and understanding of occupational therapists’ role within social prescribing.

**Method:**

Qualitative one-to-one, in depth, semi-structured interviews were carried out with occupational therapy students (*n* = 5) and educators (*n* = 4). Data were subject to framework analysis.

**Results:**

Three main themes were constructed: Knowledge of social prescribing and occupational therapy involvement, social prescribing context and education provided on social prescribing. Students had a basic knowledge of social prescribing, but there was no shared definition of social prescribing between students or educators. Students were unsure of the occupational therapist’s role within social prescribing, as they had not been exposed to this area during their practice placements.

**Conclusion:**

Universal use of the international consensus definition of social prescribing is needed to ensure consistent teaching of the approach. Including social prescribing within practice placements for occupational therapy students would aid understanding of the approach.

## Introduction and literature review

Social prescribing (SP) is a growing strategy within the United Kingdom (UK; Buck and Ewbank, 2020). According to the National Health Service (NHS), SP is *an approach that connects people to activities, groups, and services in their community to meet the practical, social and emotional needs that affect their health and wellbeing*. (National Health Service, 2021). In the UK, a range of organisations might be involved in referring people to a SP link worker to coproduce an individualised plan for support (NHS, 2021). SP is an approach that was first developed within the UK but has since gained international traction (Morse et al., 2022). Morse et al. (2022) identified 17 countries that were currently implementing SP into their healthcare systems, including Canada, the United States of America (USA), China, South Korea, Australia, the Netherlands and Portugal. Each country adapted the delivery of the approach based on population size and available resources (Zurynski et al., 2020). For example, Portugal (Hoffmeister et al., 2021), Germany (Golubinski et al., 2020), Japan (Naito et al., 2021) and Canada (Mulligan et al., 2020) have included SP duties within healthcare workers’ existing roles. Meanwhile, the UK, USA and the Netherlands developed new roles, such as link workers specifically for SP (Heijnders and Meijs, 2018; Sandhu et al., 2021; Roberts et al., 2022). Despite these worldwide developments in SP, an international consensus definition has only just emerged causing complications in generating robust evidence and international standardisation (Muhl et al., 2023).

General practitioners (GPs) are experiencing ongoing pressures within the UK’s primary care setting due to the public’s increasing demands (British Medical Association, 2023; Khan, 2023). In 2016, it was found that around 20% of patients were consulting their GP for social needs rather than medical needs (Husk et al., 2019). This has resulted in more service users waiting for appointments, issues with retaining GPs and strains on the workforce (Baird et al., 2016, 2018). The Department of Health recognised these pressures and investigated how to adapt the service to enhance patients’ independence and well-being while avoiding hospitalisation (Department of Health, 2014). As a result, the NHS stated to have made the ‘biggest investment’ by any national health service into the SP approach and included this within its long-term plan to ensure personalised care (NHS, 2021). Within the UK, a range of SP models have been used, which differs in support and intensity to adapt to the needs of the individual (Kimberlee, 2013). For example, signposting appropriate services is a low-level SP intervention, whereas allocating a link worker provides more intensive support for SP (Kiely et al., 2022). The link worker model is the most commonly used as it aims to provide a person-centred approach that is difficult to achieve by health care professionals during routine clinical consultations due to time constraints (Moffatt et al., 2017). The link worker also encourages appropriate self-help groups or social activities, such as volunteering, group learning and befriending (South et al., 2008). Many referrals are made through GPs; however, referrals can be made through a wide range of places, such as local agencies, local authorities, pharmacies, hospitals and healthcare professionals, including occupational therapists (Husk et al., 2016). Indeed, the Royal College of Occupational Therapists (RCOT) recommend occupational therapists identify local link workers and that link workers must have a clear pathway to occupational therapists (Royal College of Occupational Therapists, 2019).

The NHS proposes that the integration of SP within healthcare will alleviate the demand for primary and secondary care services (NHS, 2016), be cost-effective (Kimberlee, 2016) and provide more person-centred care (South et al., 2008). Many services and qualitative reports support the aim and trajectory of SP by describing the approach as ‘adding meaning to medicine’ (Wildman et al., 2019). However, the effectiveness of SP remains unknown as studies show mixed results (Carnes et al., 2017; Moffatt et al., 2017). A systematic review by Kiely et al. (2022), including eight studies involving 6500 participants, reported an absence of evidence for SP link workers on quality of life, mental health and healthcare costs due to high risk of bias in some of the included studies. Another review, including 15 studies, concluded that the evidence for SP link workers failed to provide sufficient details to judge either success or value for money as most were small scale and limited by poor design and reporting. Common design issues included a lack of controls, short length of follow-up, lack of standardised and validated outcome measures, missing data and failure to consider potential confounding factors leading to a high risk of bias (Bickerdike et al., 2017).

Although SP has only recently accelerated within healthcare, this style of care has been longstanding in the occupational therapist profession (Bradley and Scott, 2023) using occupational therapy theory and science (Wilcock, 2006). It has been argued that the SP concept needs a theoretical foundation to guide SP professionals’ reasoning and decision-making when supporting individuals with their needs (Ikiugu et al., 2019). Interestingly, the Person-Environment-Occupation-Performance (PEOP) occupational therapy model, which focuses on behaviour change and engagement in occupations, has been found to share similarities with SP when working towards personalised care and supporting meaningful engagement (Johansson et al., 2021). The PEOP model was found to provide a comprehensive framework that guides the process of assessing a person’s needs which is a crucial part of the SP process (Husk et al., 2019; Wildman et al., 2019) and grounded in the occupational-based framework (Johansson et al., 2021). Therefore, it is recommended that occupational therapists should be actively involved in the development of SP through the guidance of the occupational therapy theoretical framework (Johansson et al., 2021). In addition, the RCOT is seen to have taken an active involvement in the contribution that an occupational therapist can add to the SP approach including but not limited to advising on the formation of the UK National Academy for Social Prescribing and the development of an Allied Health Professionals Social Prescribing Framework (RCOT, 2019). The RCOT stated that occupational therapists are skilled at providing individualised care with an emphasis on wellness and prevention in relation to health, social and work settings (RCOT, 2019). This demonstrates a clear overlap between the role of occupational therapists and SP principles (Makanjuola, 2021); therefore, future practitioners need to build an understanding of the SP concept to support their professional identity within the approach (Thew et al., 2017). Building a professional identity within one’s role is associated with developing professional resilience and combating the blurring of roles (Ashby et al., 2016).

There is a paucity of literature exploring occupational therapy students’ understanding of SP or how their role contributes to the approach. However, various studies have explored medical students’ understanding of SP and found a lack of awareness and the need for further education (Chitson and Wylie, 2020; Mulligan et al., 2020; Santoni et al., 2019; Ward et al., 2020). This gap in the literature could be from the expectation of occupational therapists understanding SP due to shared concepts towards patient care. Meanwhile, medical students explore patients from a diagnosis angle using the medical model (Hofmann, 2005). Although this is merely an assumption, it would be insightful to explore occupational therapy students’ understanding of their role in SP. This would provide insight into student understanding of the phenomenon, education provided in SP, professional identity and student preparation for entering the workforce.

Therefore, this study aimed to investigate occupational therapy students’ perspectives and understanding of how the occupational therapist role fits within SP. This was achieved by exploring occupational therapy students’ knowledge of SP and how the role of occupational therapy contributes to the approach. Additionally, occupational therapy educators were included to gain a different perspective on the occupational therapist role in SP and the education of SP provided to occupational therapy students.

## Method

### Research design

A qualitative methodology using a phenomenological approach was chosen for this study to explore personal experiences whilst creating an in-depth understanding (Patton, 2015) of occupational therapy students’ and educators’ knowledge of the occupational therapist role within SP. This method provided rich data on lived experiences and offered participants the ability to express personal perspectives on the phenomenon (Finlay and Ballinger, 2006). The study is reported according to the consolidated criteria for reporting qualitative research checklist (Tong, Sainsbury and Craig, 2007).

### Sampling and recruitment

Participants were recruited via a combination of purposive and convenience sampling. Occupational therapy students and educators at one UK University were used as a convenience sample as the location where the lead author was undertaking further studies. Purposive sampling was used to recruit any student who was enrolled on either the Bachelor of Science (BSc) occupational therapy degree, the Master of Science (MSc) occupational therapy degree or the degree apprenticeship occupational therapist programme at one UK university and who had completed at least two practice placements, or any educators employed by the university who taught on any one of the three occupational therapy programmes (all occupational therapy professionals registered with the Health and Care Professions Council (HCPC)). Information power was used to guide sample size (Malterud et al., 2021). Information power indicates that the more information the sample holds, relevant to the actual study, the lower the number of participants which is needed. Therefore, the study initially aimed to recruit two students and one educator participant from each of the three occupational therapy programmes (*n* = 9).

Recruitment of student participants was via emailing the programme lead for each of the three programmes and requesting the lead to share the research information sheet and consent form with students. Students were asked to email the lead author to express interest and schedule an agreed time for an interview. Educators were e-mailed directly with information sheets and consent forms attached. Participants emailed completed consent forms to the lead author prior to interviews with verbal consent gained at the start of the interview.

### Data collection

Semi-structured, one-to-one interviews were conducted between 17 November 2022 and 03 February 2023 using a flexible topic guide (see Supplemental Material) with open-ended questions developed from the literature. Interviews with participants lasted up to one hour. The topic guide, exploring participants’ understanding of SP and the occupational therapist role within SP was piloted with one student and one educator. Following piloting, changes were made to the introduction, clarity of the questions and including follow-up questions. Individual interviews were chosen as this study aimed to explore participants’ narratives in-depth without the potential restrictions of a focus group (DeJonckheere and Vaughn, 2019). Participants were offered the preference of online or in-person interviews. Online interviews via Microsoft Teams were the chosen method amongst all participants as this was deemed more convenient and less time-consuming than face-to-face interviews. All interviews were carried out by the lead author who was a fellow female MSc occupational therapy student with developing experience in qualitative research techniques. The lead author was known to some of the participants as being a peer student on the MSc course and being taught by some of the educators. At the start of each interview, the lead author introduced herself as a student enrolled on the MSc occupational therapy course and provided a reminder of the research topic and purpose of the study. Data were transcribed using Microsoft Teams and audio recorded on a password-protected device to ensure the accuracy of the transcription.

### Data analysis

Framework analysis (Spencer et al., 2003) which consisted of five easy-to-follow steps suitable for a novice researcher (supervised by an experienced researcher) was used. The first step was familiarisation with all the data, followed by identifying themes throughout the transcripts, topic guide and literature in step two. Step three was to start indexing, also known as the ‘coding phase’. Step four included charting and summarising the data, with the interpretation or mapping of the data as step five. The framework used was based on the study’s aims and objectives: knowledge of SP and occupational therapist involvement, the context of SP, and education provided on SP. Data were familiarised through data collection, reading transcripts, distributing data into the framework and interpreting the data using Microsoft Excel. Once major themes, sub-themes and sub-sub-themes were generated, the second author was sent the Excel spreadsheet to ensure that analysis and interpretations were consistent and transparent.

### Ethical considerations

Ethical approval was obtained on 24 October 2022 from Northumbria University Research Ethics Committee (reference number 0123). Information sheets were distributed to students via programme leads so that students could contact the lead author to express an interest in participating. Confidentiality was guaranteed through the anonymity of participants, and the storage of anonymised data on password-protected equipment. Written informed consent was obtained from all participants before the interviews. Although the ethical review panel considered the study to be of low risk to participants, verbal consent was also obtained at the start of the interviews to ensure informed consent on the day. Debrief forms were emailed to participants after the completion of the interviews with details of the withdrawal process reiterated.

### Trustworthiness

The credibility of the data was achieved using various techniques including respondent validation. Participants were emailed raw data to validate or comment on their interview and to access trustworthiness whilst reducing researcher bias (Ahmed, 2024). The lead author also utilised reflective notetaking before and after the completion of the interviews to challenge and account for personal biases which may have influenced findings. Finally, the authors engaged as a research team to ensure appropriate record keeping, ensure interpretations of the data were consistent and transparent, and reduce researcher bias (Noble and Smith, 2015).

## Results

Interviews were carried out with five occupational therapy student participants and four occupational therapy educator participants. None of those who expressed interest refused to participate or dropped out. Participant identifying details were removed and participant numbers have been used to retain confidentiality. Participants’ characteristics are outlined in the tables below. Table 1 shows students’ characteristics. Table 2 shows educators’ characteristics.

**Table 1.** Student participants characteristics.

ID	Course	Gender	Age range	Number of placements
1	Master of Science Occupational Therapy	Female	25–34	2
2	Master of Science Occupational Therapy	Female	18–24	2
3	Master of Science Occupational Therapy	Female	25–34	2
4	Bachelor of Science Occupational Therapy	Male	25–34	2
5	Bachelor of Science Occupational Therapy	Female	18–24	4

**Table 2.** Educator participant characteristics.

ID	Years at university	Gender	Occupational Therapy Course(s)	International experience?
6	0–5 years	Male	Bachelor of Science Occupational Therapy Master of Science Occupational Therapy Occupational Therapist Apprenticeship	Yes
7	0–5 years	Female	Bachelor of Science Occupational Therapy Master of Science Occupational Therapy Occupational Therapist Apprenticeship	Yes
8	15–20 years	Female	Master of Science Occupational Therapy Occupational Therapist Apprenticeship	No
9	5–10 years	Female	Bachelor of Science Occupational Therapy Master of Science Occupational Therapy	No

**Figure 1.**
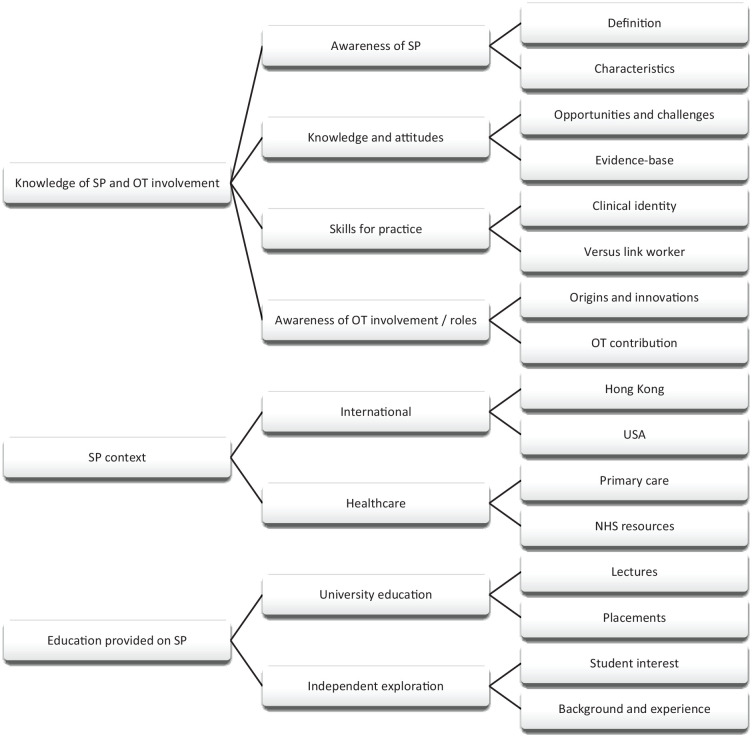
Coding tree.

Three major themes were constructed during framework analysis: (1) knowledge of SP and occupational therapist involvement, (2) SP context and (3) education provided on SP (see Figure 1).

### Knowledge of SP and occupational therapist involvement

#### Awareness of SP

All the participants showed that they understood the concept of SP to some extent. However, it was noted that they had varying levels of awareness about it, and there was no universally used definition. One of the participants pointed out the subjective nature of creating a standardised definition:

What does social prescribing actually mean. . .there’s no real definition cause it’s so subjective. . .it’s defined by the individual. (ID2)

Whilst another participant expressed the intricacies of defining the concept:

I’m sure there will be an NHS definition of what social prescribing is, but I think it’s a complex concept. (ID9)

A few participants gave their opinions on what they thought was part of SP. However, some of these answers, especially from students, were uncertain, as participant three noted:

My idea of social prescribing is there’s social prescribers. They have their own job speciality. They don’t have to be an OT, they don’t even really have to be an allied healthcare professional I don’t think. . .they signpost people towards social support or social groups that would fit with that person. And that’s the basics of what I know about it. (ID3)

Most students had a very basic understanding of the concept of SP and educators discussed the lack of awareness surrounding SP:

I probably should know more about it than I do. But maybe that’s reflective of it not being publicised. (ID7).

#### Knowledge and attitudes

One educator shared their perspective on the effectiveness of SP and noted that there is currently no scientific evidence to validate its use. The educator also mentioned that students who attempted to research the evidence base could not find substantial support for the approach:

Social prescribing is lacking of evidence-based practice. . .I also posed this question to both the degree apprenticeship students and also the BSc students in my teaching. . .we spent two sessions digging after whether social prescribing is evidence-based practice. . .sad to say both of their groups cannot take any information about evidence-based practice. (ID6).

No students could reference any literature on the topic when asked about their familiarity with SP evidence. Instead, students shared their thoughts on the potential opportunities and challenges that SP could face. Some examples discussed were how SP could support individuals dealing with loneliness and mental health issues and how it could be a cost-effective primary care solution. However, concerns were raised about the need for more funding, awareness and GP practices offering this approach.

#### Skills for practice

Participants also discussed the role of occupational therapists and how their specialised training and skills could contribute to SP, potentially differing from that of a link worker. This could include dual training in mental and physical health, conducting assessments, using grading techniques and providing environmental adaptations. However, some students expressed uncertainty about distinguishing between the two roles, as they lacked experience in this setting or with link workers:

I’m just guessing here and I might be wrong. . .with an occupational therapist we’ve got specific training which may be a bit more advanced than what a link worker is given, for example in the depth and breadth of assessments that we can do and our focus on environmental factors, our training like grading and adaptations and for activities. So, we’ve got that knowledge. . .I don’t know enough about social prescriber’s roles to know if they get that same training as well. (ID1).

All participants shared their thoughts on the importance of clinical identity in the occupational therapist profession. The students spoke about their difficulties developing a professional identity as new practitioners. It was identified that the role of the occupational therapist can be seen as being very ambiguous to outsiders, especially when explaining the variations in the role:

You don’t have to be an occupational therapist to be a social prescriber, I suppose it’s like . . . does it deskill us? . . . even just . . . the concept of our role is quite vague or it’s hard to get your head around. (ID2)

Educators appreciated the difficulties students might face as newly graduated occupational therapists ‘*I think it’s tricky’ (ID9)* but were confident in students’ abilities and knowledge to create a sense of professional identity within the workplace. Educators spoke about this as a positive challenge rather than something to fear:

It would be a challenge . . . I don’t think it’s any more of a challenge than establishing your own professional identity wherever you work. . .in fact, it could even be a real opportunity for reinforcing and strengthening your professional identity because you would be the only advocate within that. (ID8)

#### Awareness of occupational therapist involvement

It was emphasised that the connection between occupational therapy and SP *links beautifully (ID7)*, and it was reported to be *an absolute perfect fit (ID8).* Educators highlighted that SP was used within occupational therapy before the creation of SP within healthcare. This was discussed through clinical experiences before SP and how occupational therapists naturally connected people to their home environments and communities:

We’ve been doing social prescribing since the inception of occupational therapy, it’s what we do. We use a range of different types of activities both in the community that we utilise with people. (ID8)

Participants shared their thoughts on whether they considered SP an innovative approach. While everyone agreed that the approach shares similar concepts with occupational therapists, opinions on its level of innovation varied. A couple of participants saw SP as highly innovative:

I think it is innovative in that sense because it’s saving them money. It’s coming up with a new way to do things. (ID4)

Whereas other participants were more reluctant to define SP as truly innovative:

I think it’s innovative in terms of where it’s sitting within primary care. . .it’s not that innovative, it’s just been given a shiny new relaunch by the government. (ID8)

This belief that SP had simply been ‘relaunched’ was echoed by participant one who described it as a *sort of repackage . . . cheaper way* to occupational therapy.

### SP in context

#### International

Only a few individuals mentioned SP and occupational therapy in a particular context. Specifically, two educators highlighted the disparities in international healthcare and noted that SP is not utilised in some countries. Additionally, one participant shared their observations on the differences in the healthcare system in Hong Kong:

Social prescribing . . . is new to me because in Hong Kong we haven’t had this type of model . . . in Hong Kong we got a . . . social welfare department . . . the hospital authority in Hong Kong only caters for the medical and health related issue. (ID6)

Furthermore, another participant discussed the healthcare system in the USA and how its priorities differ from those of the UK:

And because I was working in a very defined way . . . you have very clear goals and they have to be really measurable, achievable, and actually something like social prescribing wouldn’t have been a priority for the insurers. Whereas grip strength or range of motion, or something very tangible would. (ID7)

#### Healthcare

Some participants spoke about SP within the UK primary care setting. A student cited that the RCOT advocates for occupational therapists to be placed within primary care and how this may support SP at the coalface rather than secondary care:

I was reading recently about occupational therapy in primary care and that’s a big push for the RCOT and one of their research priorities. I wonder how that links in with social prescribing, because obviously, that is a place where they’re trying to push for more from an occupational therapist who could do that social prescribing role. (ID1)

This topic included educators discussing NHS resources and the funding available:

. . .social prescribing is occupational therapy on the cheap . . . for a GP practice to employ a full-time occupational therapist you would need band 5 and band 6 to do it effectively. Then your link workers who would work as your OT assistant, and it’s expensive. And the whole point of this is to save money. But nothing good ever comes from saving money . . . you’re throwing all this money at it, and what you have wasted is that golden opportunity of using the very people that the NHS trains . . . who already have that knowledge. (ID8)

Educators discussed how making use of existing occupational therapist skills could be a better use of scarce NHS resources specifically considering the lack of evidence for SP.

### Education provided on SP

#### University education

All participants stated there were lectures provided to students around SP. Most students said they had not known about the concept before attending university. However, there was a split opinion on whether enough teaching was provided around SP and if there had been enough discussions of how the occupational therapist role fits within the concept. Educators stated that all the occupational therapy programmes had the same teaching in SP. Additionally, role-emerging placements allowed students to experience working in environments without occupational therapists but where they could have an SP role:

We have one placement that we’ve just set up where the OT, it would be recognised as having a more managerial social prescribing role. (ID9).

However, during interviews with students, it was discovered that many were unfamiliar with the concept of a role-emerging placement for SP.

#### Independent exploration

Educators noted that a student’s background and experiences can impact their understanding of the concept and how it relates to occupational therapy. Educators emphasised the importance of maturity and real-life experiences in comprehending and applying the SP concept:

It might be a little bit easier for those more mature students. Maybe they’ve had some more experience of working in different settings. (ID7).

In addition, educators stated that while they could facilitate discussions around the concept of SP until students had experienced SP themselves, it was very difficult to teach in a classroom setting.

## Discussion and implications

This study investigated occupational therapy students’ perspectives and understanding of the occupational therapist role within SP. This included speaking to occupational therapy students and occupational therapy educators for insight into education on SP and their knowledge of the occupational therapist role within SP. Three main themes were constructed from the interviews: knowledge of SP and occupational therapist involvement, SP context and education on SP. Findings revealed that students had a basic awareness of SP concepts through university education. However, the students appeared unsure of the occupational therapist role within the SP setting, as they stated they had not been exposed to this area during their practice placements.

Throughout the knowledge theme, it was recognised that there was no shared definition of SP between students or educators, with many respondents displaying a lack of confidence when asked to provide a definition. However, the participants were able to express similar characteristics of SP, including non-medicalised interventions, link worker involvement, linking to the community and supporting an individual’s loneliness and social needs. Interestingly, the difficulty with conceptualising a definition of SP is also shared within the literature (Husk et al., 2019). As SP is used differently regionally, nationally and internationally, this has implications for generalising the concept (Husk et al., 2019). Internationally accepted conceptual and operational definitions of SP have recently been developed via expert consensus; however, these are yet to be widely used (Muhl et al., 2023). This uncertainty in defining and understanding SP may impact teaching the concept to students, especially if the literature and university educators are currently unable to define the concept themselves.

Students reported that they had not heard of SP until the university provided education about the approach. However, the students felt they could not put theory into practice as they had not experienced SP during a practice placement. This identified a gap within student learning, as it is suggested that one’s professional identity starts in the classroom and develops through practical experience (Clarke et al., 2015). The educators’ mentioned SP was within some of the practice placements, known as role-emerging placements, but not all students would have this opportunity. As the students within the study stated they had no exposure to the occupational therapist role within SP, they could only assume what the occupational therapist role may contribute to the approach.

Nevertheless, students reported that occupational therapists could provide specific skills and knowledge, such as assessments and occupational therapy theory, to offer a deeper level of support for individuals compared to link workers. Exposing students to settings within grey areas, such as SP, encourages the development of professional identity by allowing them to generate a vision of a therapist they aspire to be within an ever-changing health and social care climate (Clarke et al., 2015). One study has explored SP’s relevance within the occupational therapist role, with qualified occupational therapists practising in an NHS secondary care community setting (Truman, Chamberlain and Pike, 2019). The occupational therapists expressed the opportunity SP offered for them to utilise their core skills whilst working towards their professional vision. However, these occupational therapists had already created a foundation for their clinical identity and could understand their role in a developing area contrary to the occupational therapy students within this study, who are at the early stages of exploring clinical identity and understanding the differences and similarities between the occupational therapist and SP roles.

A similar study in the UK explored medical students’ awareness of SP from 27 different medical schools (Mulligan et al., 2019). The study provided students with surveys before and after SP lectures and received over 900 responses. It was found that the students had heard of the critical components of SP but needed to be made aware of the term. Thus, reinforcing a need for broader education and a universally used definition. The study identified potential barriers to GPs including SP within their care. Some of the most common challenges were needing more time within consultations and forgetting to offer SP as an option (Pescheny et al., 2018). This limited awareness highlighted a need to include SP in medical training to prepare and equip future doctors to integrate SP within patient care (Santoni et al, 2019). Mulligan et al.’s (2019) study shared similarities with the present research exploring UK students’ understanding of SP. The results identified a surface-level awareness of the concept in both medical and occupational therapy students. The differences arose in the type of students the studies examined, and the methodology used to explore the participants’ narratives. Mulligan et al. (2019) used a quantitative method to explore a large sample size of students. They utilised a before and after survey to examine the education provided for medical students. In contrast, this study investigated a small number of students/educators in depth. Both studies highlight a need for continuing SP education in healthcare professionals and how this can be used within their roles.

## Strengths and limitations

This study explored a new angle in SP by examining students from an occupational therapist background, thus, adding to the existing literature. The sample provided variation in participants by exploring occupational therapy students across different programmes and incorporating the educator’s viewpoints for an in-depth understanding of SP and occupational therapy. However, there was no participation from apprenticeship students due to issues with placement commitments. Therefore, the results cannot portray apprenticeship students’ understanding of the phenomenon, thus, leaving a gap in knowledge for this group. Second, this study contained a relatively small sample size which would prevent generalisation of the data. The study was also limited to one university; therefore, results may differ in other locations around the UK. Third, a novice researcher conducted the study, revealing challenges relating to a lack of expertise and experience. These challenges arose when conducting interviews and reporting the research project results. The limitation of being a novice researcher was mitigated as much as possible through close supervision by an experienced researcher. Finally, framework analysis was carried out by the lead author with interpretation of the data discussed between authors. This lack of independent coding could have introduced bias into the results.

## Conclusion

There is a need within the literature to use the international consensus definition of SP and to ensure that the teaching of SP is consistent in using universal terminology. Including SP within at least one practice placement would enable students to fully encompass the SP concept and build an occupational therapist identity before entering the workforce. Further research could explore occupational therapy students’ understanding of SP on a larger scale, nationally and internationally.

**Key findings**

- Conceptualising social prescribing was difficult for students and educators.
- Students were found to have a basic awareness of social prescribing.
- Students had not been exposed to social prescribing during their practice placements.

**What the study has added**

There remains a need for the universal use of the international consensus definition of social prescribing to ensure that teaching of social prescribing is consistent. It may be beneficial to include SP in at least one practice placement.

## Supplemental Material

sj-docx-1-bjo-10.1177_03080226241270442 – Supplemental material for Occupational therapy students’ and educators’ perspectives and understanding of the role of occupational therapy within social prescribing: A qualitative interview study Supplemental material, sj-docx-1-bjo-10.1177_03080226241270442 for Occupational therapy students’ and educators’ perspectives and understanding of the role of occupational therapy within social prescribing: A qualitative interview study by Siobhan Elliott and Catherine Haighton in British Journal of Occupational Therapy
