# Supplementary material for: Occupational therapy students’ and educators’ perspectives and understanding of the role of occupational therapy within social prescribing: A qualitative interview study
Source: Br J Occup Ther. 2024 Aug 13;88(1):35–44. doi: 10.1177/03080226241270442 (PMC11887886; doi:10.1177/03080226241270442)
Supplement: sj-docx-1-bjo-10.1177_03080226241270442 – Supplemental material for Occupational therapy students’ and educators’ perspectives and understanding of the role of occupational therapy within social prescribing: A qualitative interview study [file sj-docx-1-bjo-10.1177_03080226241270442.docx]

**Supplementary material**

**Topic guide**

What is Pre-registration BSc, MSc, and apprenticeship occupational therapy (OT) students’ perspectives and understanding of the OT role within social prescribing?

***SP description for participants if needed.***

Social prescribing enables healthcare professionals (often GP’s) to refer service users to a link worker who uses a holistic approach to connect people with local, non-clinical community services (e.g., befriending, volunteering, cooking groups etc). OT’s can refer to a link worker, however, this may not be required due to their knowledge and expertise. This concept is designed to support people with a range of social, emotional, or practical needs with a focus on health and well-being. With an aim to prevent hospitalisation and provide strategies to remain well in the community.

***Interview topic guide questions***

***Student questions:***

1. Demographics

- Age?
- Gender?
- What course?
- Which programme? BSc, MSc, Apprenticeship?

1. How many practice placements have you completed? And could you describe what your current practice placement experience is?
2. Did you have healthcare experience before starting this course?

- If yes, can you tell me what your previous experience was?

1. What is your knowledge of social prescribing?

- No? **Provide a brief written description of SP.
  - After reading this description, do you think you heard of it?
- What would you say are the aims/benefits of SP?

1. Do you know anything about the evidence base around social prescribing?

- Are there any debates around the evidence to prove this is an effective approach?

1. Is there a difference in roles between a link worker and OT?

- Yes/no – can you expand?
- How would a client/healthcare professional know the difference in roles?

1. What do you think the role of an OT could offer to the SP scheme?

- Are there any skills/core values that an OT could use? If so, what are they?

1. Do you think SP is an innovative scheme?

- Yes/no – why?
- Is there any relation to the OT?

1. How would you feel about promoting/working with the SP scheme when graduating?

- Can OTs work in SP? If so, how?
- Would you feel confident in your professional identity in SP? Why?
- Would this be an area you would like to work in? why?

1. What information has the University provided about SP?

- Have you had lectures? What did this include?
- No – do you think this should be included in the curriculum?
- Yes – was this lecture enough?
  - was there a discussion around the role of OT in SP, if so, what was the discussion?

1. Would you like further information about SP?

- What would this look like? E.g., provided in placements/lectures?
- No? why? Is it irrelevant, lacks interest, or has been taught enough?

1. Is there anything you would like to add further to the OT role in SP?

***Educator questions****:*

1. Demographics

- Age?
- Gender?
- What programme do you teach?
- Which students does this include? (BSc, MSc, app)

1. Could you tell me how long you have worked as a lecturer/at this University? And describe your work experience as a lecturer.
2. What was your work experience before lecturing? And could you describe this experience?

- Were you an OT? Worked in healthcare?

1. What is your knowledge of social prescribing?

- No? **provide a brief written description of SP.
  - After reading this description, do you think you may have heard of it?
- What would you say are the aims/benefits of SP?

1. Is there a difference in roles between a link worker and OT?

- Yes/no – can you expand?
- How would a client/healthcare professional know the difference in roles?
- Is the role of a link worker/OT interchangeable? E.g., qualifications, experience, skills?

1. What do you think the role of an OT could offer to the SP scheme?

- Are there any skills/core values that an OT could use? If so, what are they?

1. Do you think SP is an innovative scheme?

- Yes/no – why?
- Is there any relation to the OT role?

1. How do you think students would feel about promoting/working with the SP scheme when graduating?

- How can OTs contribute/work within SP?
- Do you think they would have enough knowledge/experience to promote/work in the field? Why?
- Do you think they would feel confident in their professional identity? Why?
- Do you think this is an area students would like to work in? e.g., why?

1. What information has the University provided about SP?

- Have you had lectures? What did this include?
- No – do you think this should be included in the curriculum?
- Yes – was this lecture enough?
  - was there a discussion around the role of OT in SP, if so, what was the discussion?

1. Would you like further information about SP?

- What would this look like? E.g., provided in placements/lectures?
- No? why? Is it irrelevant, lacks interest, or has been taught enough?

1. Is there anything you would like to add further to the OT role in SP?
